# The Exposure of Breast Cancer Cells to Fulvestrant and Tamoxifen Modulates Cell Migration Differently

**DOI:** 10.1155/2013/147514

**Published:** 2013-07-02

**Authors:** Dionysia Lymperatou, Efstathia Giannopoulou, Angelos K. Koutras, Haralabos P. Kalofonos

**Affiliations:** Clinical Oncology Laboratory, Division of Oncology, Department of Medicine, University of Patras, Patras Medical School, 26504 Rio, Greece

## Abstract

There is no doubt that there are increased benefits of hormonal therapy to breast cancer patients; however, current evidence suggests that estrogen receptor (ER) blockage using antiestrogens is associated with a small induction of invasiveness *in vitro*. The mechanism by which epithelial tumor cells escape from the primary tumor and colonize to a distant site is not entirely understood. This study investigates the effect of two selective antagonists of the ER, Fulvestrant (Fulv) and Tamoxifen (Tam), on the invasive ability of breast cancer cells. We found that 17**β**-estradiol (E_2_) demonstrated a protective role regarding cell migration and invasion. Fulv did not alter this effect while Tam stimulated active cell migration according to an increase in Snail and a decrease in E-cadherin protein expression. Furthermore, both tested agents increased expression of matrix metalloproteinases (MMPs) and enhanced invasive potential of breast cancer cells. These changes were in line with focal adhesion kinase (FAK) rearrangement. Our data indicate that the anti-estrogens counteracted the protective role of E_2_ concerning migration and invasion since their effect was not limited to antiproliferative events. Although Fulv caused a less aggressive result compared to Tam, the benefits of hormonal therapy concerning invasion and metastasis yet remain to be investigated.

## 1. Background

Breast cancer is the most frequent malignancy cancer in women. It is estimated that approximately 75% of breast tumors are estrogen receptor (ER) positive, and their growth is stimulated by estrogens [1]. Estrogen-based therapies represent the mainstay in the treatment of hormone-dependent breast cancer with the ER modulator Tamoxifen (Tam) improving significantly the clinical outcome of patients with both early and advanced breast cancer [2]. Furthermore, Fulvestrant (Fulv) that belongs to a recently developed group of antiestrogens (selective estrogen receptor downregulators—SERDs) has extended the therapeutic options in the management of breast cancer patients [2, 3].

Invasion is considered as the hallmark of malignancy and is the first in the cascade of events leading to tumor development and metastasis. During invasion, the tumor cells penetrate into tissues breaking the basement membrane and allowing tumor growth. The invading tumor cells are able to enter the circulation so as distant metastasis occurs [4, 5]. Both invasion and metastasis require cell migration. The cell type and tissue microenvironment define the way of cell movement that is generally categorized as single and collective cell migration. During single cell migration, cells disseminate from the primary tumor as individual using either amoeboid or mesenchymal type movement, while in collective migration cells move as cell sheets or clusters [6, 7].

Degradation of the extracellular matrix (ECM) is one of the most important events in the spread of malignant cells, and it is well documented that it plays an essential role in tumor prognosis [8]. Matrix metalloproteinases (MMPs), zinc finger dependent enzymes, promote invasion, metastasis, and angiogenesis through the digestion of ECM components as well as surface factors' receptor and junctional proteins involved in cell-cell and cell-ECM interactions. MMPs consist of 23 members, which are classified into different groups, including gelatinases. MMP-2 and MMP-9 are gelatinases that are related to tumor invasion and metastasis by their capacity for tissue remodeling via ECM, as well as their involvement in epithelial mesenchymal transition (EMT) [8, 9]. EMT is the key mechanism by which tumor cells gain invasive and metastatic ability, as EMT enables separation of individual cells from the primary tumor mass and promotes cell migration. During EMT, epithelial cells lose polarity and cell-cell contacts and undergo a complete remodeling of the cytoskeleton that leads to the acquisition of the mesenchymal features such as motility, invasiveness, and resistance to apoptosis [10–12]. One of the most pivotal steps in this process is the loss of E-cadherin, a cell-adhesion protein that maintains the cell-cell contacts [13]. However, the expression of E-cadherin is regulated by several transcription factors including Snail, Slug, and Twist. Furthermore, the nonreceptor tyrosine kinase focal adhesion kinase (FAK) is associated with highly invasive breast cancers, and it mediates several pathways leading to proliferation, migration, and adhesion [14]. Phosphorylation is required for FAK activation, and it has been shown that estrogens are able to promote rapid phosphorylation of FAK at tyrosines residues [15].

Despite the undoubted benefits that estrogen-based therapies offer to ER^+^ breast cancer patients, *de novo* and acquired resistance to such therapies presents a major clinical problem [16]. The aim of the current study is to evaluate the effect of antiestrogens Fulv and Tam as well as the active metabolites of Tam, Endoxifen (End), and 4-OH-Tamoxifen (4-OH-T) on migration of 17*β*-estradiol- (E_2_-) stimulated breast cancer cells. We focused on single and collective cell migration since these are the main ways for cells to migrate. To understand the effect of estrogen receptors' inhibition on cell migration, we assessed the effect of the antiestrogens on MMPs levels, on protein levels as well as on localization of E-cadherin and Snail and colocalization of FAK phosphorylated form with actin fibers.

## 2. Methods

### 2.1. Cell Culture and Reagents

In the current study, the human hormone-dependent breast cancer cell lines MCF-7 and T47D were purchased from the American Type Culture Collection (ATCC, USA). The adenocarcinoma cell line MCF-7 was cultured in EMEM supplemented with 2 mM L-glutamine, 0.1 mM nonessential amino acids, and 10% fetal bovine serum (FBS). The ductal carcinoma cell line T47D was cultured in RPMI 1640 supplemented with 4.5 g/L glucose (Sigma-Aldrich, Inc., USA) and 10% FBS. Both mediums were supplemented with 0.01 mg/mL insulin (Sigma-Aldrich, Inc., USA), 1 mM sodium pyruvate, 1.5 g/L sodium bicarbonate, 100 *μ*g/mL penicillin G/streptomycin, 2.5 *μ*g/mL amphotericin B, and 50 *μ*g/mL gentamycin. All mediums and supplements were purchased from Biochrom (Berlin, Germany) unless otherwise indicated. Cells were cultured at 37°C, 5% CO_2_, and 100% humidity.

E_2_, Fulv, Tam, End, and 4-OH-T were purchased from Sigma-Aldrich (Sigma-Aldrich, Inc., USA). All experiments were performed according to the following conditions: after reaching 70% confluence, cells were washed with phosphate buffer saline (PBS) and incubated with phenol red-free RPMI (rf-RPMI) (Biochrom, Berlin, Germany) with 1% charcoal-stripped serum (CSS) for 24 h to deplete estrogen [17]. Thereafter, cells were treated with E_2_ and the tested agents at the indicated time points and doses according to appropriate assay.

### 2.2. Cell Proliferation Assay

The effect of E_2_ and the tested agents on proliferation of cells was determined using the 3-(4,5-dimethylthiazol-2-yl)-2,5-dephenyltetrazolium-bromide (MTT) assay, as previously described [18]. Briefly, both MCF-7 and T47D cells, were seeded at a density of 2 × 10^4^ cells/well in 24-well plates with rf-RPMI supplemented with 1% CSS. Cells were treated with E_2_ 10 nM alone or in combination with the tested agents: Fulv + E_2_, Tam + E_2_, End + E_2_, and 4-OH-T + E_2_ for 48 h. The tested agents were added at two different concentrations: 100 nM and 1 *μ*M. MTT solution (5 mg/mL in PBS) was prepared and a volume equal to 1/10 was added to each well and incubated for 2 h, at 37°C. Medium was removed and 100 *μ*L acidified isopropanol (0.33 mL HCl in 100 mL isopropanol) was added in each well in order solubilise the dark blue formazan crystals. The solution was transferred to 96-well plates and immediately read in a microplate reader (Tecan, Sunrise, Magellan 2) at a wavelength of 570 nm using reference wavelength 620 nm.

### 2.3. Migration Assay

Migration assay was performed using boyden chambers (Costar, Avon, France) containing uncoated polycarbonate membranes with 8 *μ*m pores. Briefly, cells were treated with E_2_ and the tested agents for 24 h with rf-RPMI supplemented with 1% CSS. Cells were trypsinized and resuspended at 2 × 10^4^ cells/0.1 mL in the same medium in presence of E_2_ and the tested agents. The bottom chamber was filled with 0.6 mL of rf-RPMI with 10% CSS. The upper chamber was loaded with the solution of 2 × 10^4^ cells and incubated for 36 h. After incubation, the membrane was fixed with saline-buffered formalin and stained in 1% toluidine blue solution. Images of cells that have migrated through the filter were captured using an inverted microscope of Nikon (Eclipse TE 2000-U) at magnification of 10X.

### 2.4. Invasion Assay

To evaluate the effect of E_2_ and tested agents on capacity of cell to invade, a Boyden chamber containing matrigel-coated polycarbonate membranes with 8 *μ*m (Invasion Chambers, BD Biosciences, Oxford, UK) was used. Briefly, cells were treated with E_2_ and the tested agents for 24 h with rf-RPMI supplemented with 1% CSS. Cells were trypsinized and resuspended at 1.25 × 10^5^/mL in the same medium in presence of E_2_ and the tested agents. The bottom chamber was filled with 0.7 mL of rf-RPMI with 10% CSS. The upper chamber was loaded with the solution of 1.25 × 10^5^cells and incubated for 72 h at 37°C. After the incubation, the noninvading cells were removed from the upper compartment using a cotton swab. Transwell filters were fixed with saline-buffered formalin for 10 min and then in 100% methanol for 20 min. Cells were stained in toluidine blue solution for 10 min and washed twice in 1% PBS. Images of cells that have migrated through the matrigel-coated filter were captured using an inverted microscope of Nikon (Eclipse TE 2000-U) at magnification of 10X.

### 2.5. Scratch-Wound Assay

The effect of E_2_ and the tested agents on collective cell migration was evaluated using 2D scratch-wound assay. Briefly, cells were seeded in 6-well plates at a density of 10^5^ cells/well. After reaching 100% of confluence, cells were treated with E_2_ and the tested agents in the appropriate medium rf-RPMI with 10% CSS for 24 h. In the confluent cells' monolayer an artificial gap was created with a yellow pipette tip. Then cells were rinsed several times with the appropriate medium to remove dislodged cells. Images of living cells were captured at the indicated time points of 0, 24, and 48 h at magnification of 4X using an inverted microscope (Nikon Eclipse TE 2000-U).

### 2.6. Zymography

Zymography was used to evaluate the expression both of pro- and active forms of MMP-2 and MMP-9. Supernatants from both cell lines were collected in 48 h, concentrated 80-fold to 50 *μ*L, and analyzed as previously described [18].

### 2.7. Immunoblotting

E-cadherin and Snail were studied using western blot analysis. Briefly, MCF-7 and T47D cells were treated with E_2_ and the tested agents for 24 and 48 h, and then cells were lysed in buffer containing 0.5% NP-40, 0.5% NaDOC, 0.1% SDS, 50 mM Tris (pH 7.0), 150 mM NaCl, 1 mM EDTA (pH 8.0), 1 mM NaF, and a protease inhibitor cocktail (Sigma-Aldrich, Inc., USA), as previously described [19]. Cell extracts were incubated on ice for 30 min, with vortexing every 10 min and centrifuged at 13000 rpm for 30 min. Supernatants were collected and protein concentration was determined with Bradford (Sigma-Aldrich, Inc, USA) assay. Specific protein amount was analyzed using the standard procedure of western blot analysis. A mouse anti-E-cadherin (1 : 1000, Invitrogen Corporation, Camarillo, CA, USA), a rat anti-Snail (1 : 1000, Cell Signaling Technology, Inc., Boston, USA), and a mouse antiactin (1 : 1000, Chemicon, Millipore, Temecula, CA, USA) were used. Detection of the immunoreactive proteins was performed by chemiluminescence using horseradish peroxidase substrate SuperSignal (Pierce, Rockford, IL, USA), according to the manufacturer's instructions.

### 2.8. Immunofluorescence

Cells were grown in 4-well coverslips (15 × 10^3^ cells/well) in the presence or absence of E_2_ and the tested agents for 48 h. Cells were fixed with saline-buffered formalin for 15 min and permeabilized with 0.1% Triton for 5 min. Blocking was performed with 3% bovine serum albumin (BSA) in phosphate buffer saline (PBS) containing 10% FBS for 1 h at 37°C. After the incubation, cells were rinsed once with PBS for 5 min and then incubated with a mouse anti-E-cadherin (1 : 1000, Invitrogen Corporation, Camarillo, CA, USA), a rat anti-Snail (1 : 500, Cell Signaling Technology, Inc., Boston, MA, USA), a rabbit anti-Tyr^397^-FAK antibody (dilution 1 : 200, R&D Systems, Deutschland, Germany), a mouse anti-ER-*α* antibody (dilution 1 : 500, Chemicon International Inc., Temecula, CA, USA), and phalloidin-fluorescein isothiocyanate labeled (Sigma-Aldrich, Inc., USA) for 1 h at 37°C. Cells were rinsed 3 × 5 min with PBS and then a chicken anti-mouse Alexa Fluor 488, a chicken anti-rat Alexa Fluor 568, or a donkey anti-rabbit antibody Alexa Fluor 594, (1 : 1000, molecular probe, Invitrogen Corporation, Camarillo, CA, USA) diluted in blocking solution and an incubation for 30 min at 37°C was followed. Cells were rinsed 2 × 5 min with PBS; then incubation for 5 min with 5 *μ*M Draq 5 (Biostatus Limited, Shepshed, UK) or DAPI (Vectashield, Vector Laboratories, Inc., US) diluted in PBS was followed for nucleus staining and cells mounted on glass sides. Fluorescence was visualized using a Leica microscope at 63X magnification.

### 2.9. Statistical Analysis

Differences between groups and controls were tested by one-way ANOVA. Each experiment included at least triplicate measurements. All results are expressed as mean ± SEM from at least three independent experiments.

## 3. Results

### 3.1. Fulv, Tam, and the Metabolites End and 4-OH-T Partially Decrease E_2_-Induced Cell Proliferation

**Figure 1:**
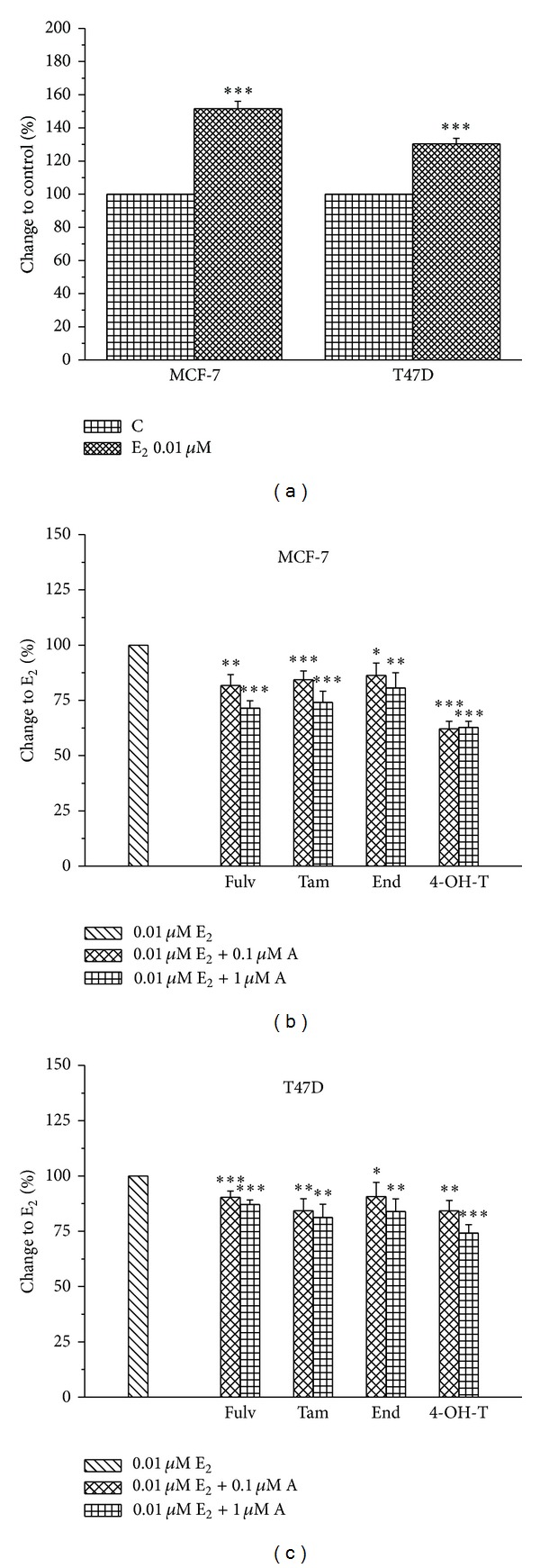
The effect of E_2_ and the tested agents on cell proliferation. E_2_ alone induces cell proliferation of MCF-7 and T47D (a). Cells were pretreated with E_2_ (0.01 *μ*M), and the tested agents (A) were added at the concentrations of 0.1 and 1 *μ*M at MCF-7 (b) and T47D (c). Results are expressed as mean ± SEM of the % change compared to the untreated cells and/or E_2_. Asterisks denote a statistically significant difference compared to control (untreated) cells. *P < 0.05, **P < 0.01, and ***P < 0.001.

In the current study, MCF-7 and T47D breast cancer cells were treated with Fulv, Tam, and its metabolites End and 4-OH-T, so as to determine the optimum concentration regarding their effect on cell proliferation. E_2_ was used at a concentration of 0.01 *μ*M as previously described [20]. Fulv, Tam, End, and 4-OH-T were tested at the concentrations of 0.1 and 1 *μ*M, as previously described [21–24]. Both cell lines were treated with Fulv and Tam as well as the metabolites of the latter with simultaneous addition of E_2_. We showed that E_2_ induced cell proliferation in both cell lines 48 h after its addition (Figure 1(a)), as previously described [25]. All the tested agents demonstrated an antiproliferative effect in both concentrations in a dose-dependent manner compared to untreated cells in both cell lines 48 h after their addition, as was expected (Figures 1(b) and 1(c)). Thereafter, all the experiments were performed using 0.01 *μ*M E_2_ and 0.1 *μ*M of the tested agent.

### 3.2. Tam but Not Fulv Stimulates Single Cell Migration

**Figure 2:**
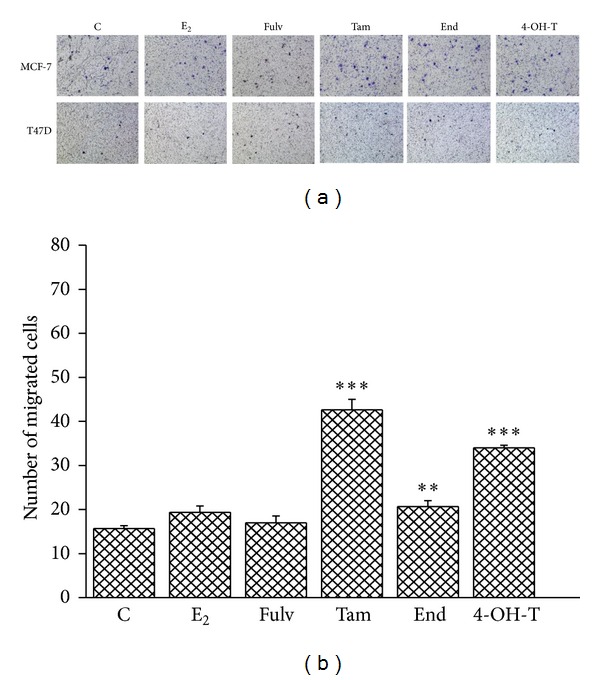
Single cell migration in MCF-7 and T47D cells after their treatment with E_2_ and antiestrogens. C: control (untreated cells); E_2_: cells treated with 17*β*-estradiol; Fulv: cells treated with E_2_ + 100 nM Fulv; Tam: cells treated with E_2_ + 100 nM Tam; End: cells treated with E_2_ + 100 nM End; and 4-OH-T: cells treated with E_2_ + 100 nM 4-OH-T. The image is representative of three independent experiments using a magnification of 10X (a). Quantification of images from boyden chamber assay in MCF-7 cells (b). Results are expressed as mean ± SEM of the % change compared to the untreated cells. Asterisks denote a statistically significant difference compared to E_2_ treated cells. **P < 0.01 and ***P < 0.001.

Migration is a pivotal process for both invasion and metastasis allowing cells to change position into tissues or metastasize to distant organs [5, 26]. Cancer cells utilize different ways to migrate, either individual or multicellular [4]. To assess the effect of the tested agents on single cell migration, we used the boyden chamber assay in both cell lines. Cells were pretreated with E_2_ and the tested agents for 24 h, and then we observed their ability to migrate through the membrane after 36 h incubation. MCF-7 cells showed greater ability to pass through the membrane compared to T47D cells (Figure 2). E_2_ alone or in combination with Fulv did not affect MCF-7 cell migration compared to untreated cells. In contrast the treatment of MCF-7 cells with the combination of E_2_ with Tam and its metabolites significantly promotes the motility of cells to migrate through the pores of the membrane (Figure 2). In T47D cells the effect of E_2_ and the tested agents on cell migration is not reliable since very low number of cells passed through the membrane. The difference in the ratio of ER*α*/ER*β* might contribute to low metastatic ability of T47D cells. MCF-7 cells express very low levels of ER*β* compared to T47D cells [27]. According to recent data, ER*β* exerts a protective role for the cell by inhibiting the invasiveness and promoting the adhesion [28]. Further, a previous study demonstrated that treatment of MCF-7 cells with E_2_ caused a degradation of ER*α* and an increase of ER*β* [29]. This might explain the absence of any effect on MCF-7 cell migration after their treatment with E_2_ alone or in combination with Fulv since Fulv exerts its effect through ER*α* degradation.

### 3.3. Collective Cell Migration Is Not Affected by Fulv but It Is Reduced by Tam

**Figure 3:**
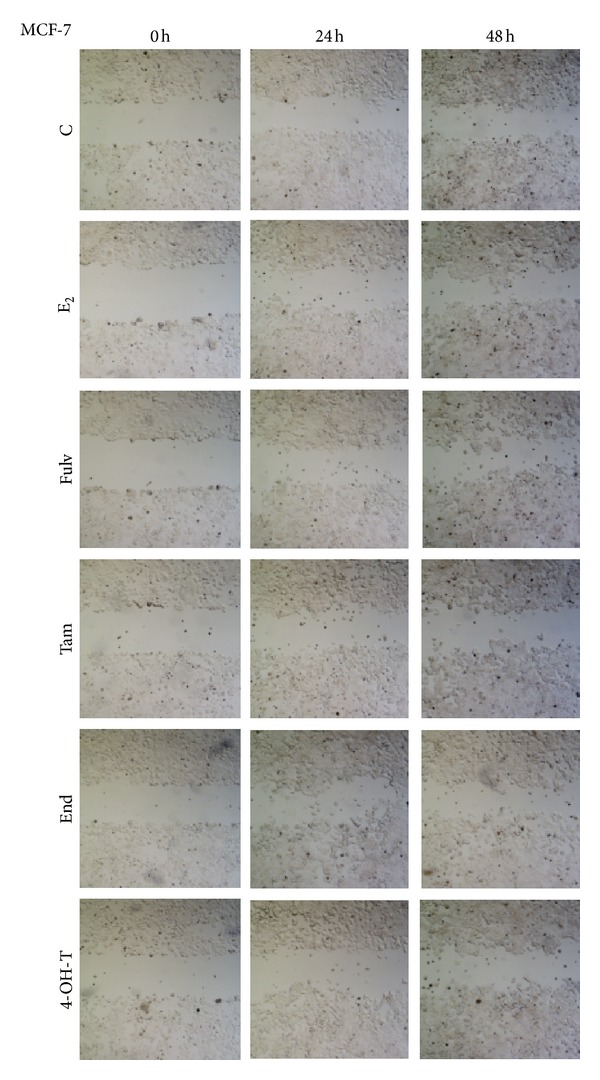
Collective cell migration in MCF-7 cells treated with E_2_ and antiestrogens. C: control (untreated cells); E_2_: cells treated with 17*β*-estradiol; Fulv: cells treated with E_2_ + 100 nM Fulv; Tam: cells treated with E_2_ + 100 nM Tam; End: cells treated with E_2_ + 100 nM End; and 4-OH-T: cells treated with E_2_ + 100 nM 4-OH-T. The image is representative of three independent experiments using a magnification of 4X.

Since E_2_ alone or in combination with Fulv did not affect single cell migration, we studied the effect of tested agents on collective cell migration using the scratch wound assay [30]. Both cell lines were treated with E_2_ and the tested agents for 24 and 48 h. In MCF-7 cells we found that E_2_ alone increased cell migration compared to untreated cells up to 48 h (Figure 3). The combination of E_2_ with Fulv reversed slightly the effect of E_2_ alone. This reversal was more potent when E_2_ combined with Tam, End, and 4-OT-T as shown in Figure 3. The same effect of E_2_ and tested agents was observed in T47D (data not shown).

### 3.4. Fulv and Tam Totally Reverse the Protective Effect of E_2_ in Cell Invasion

**Figure 4:**
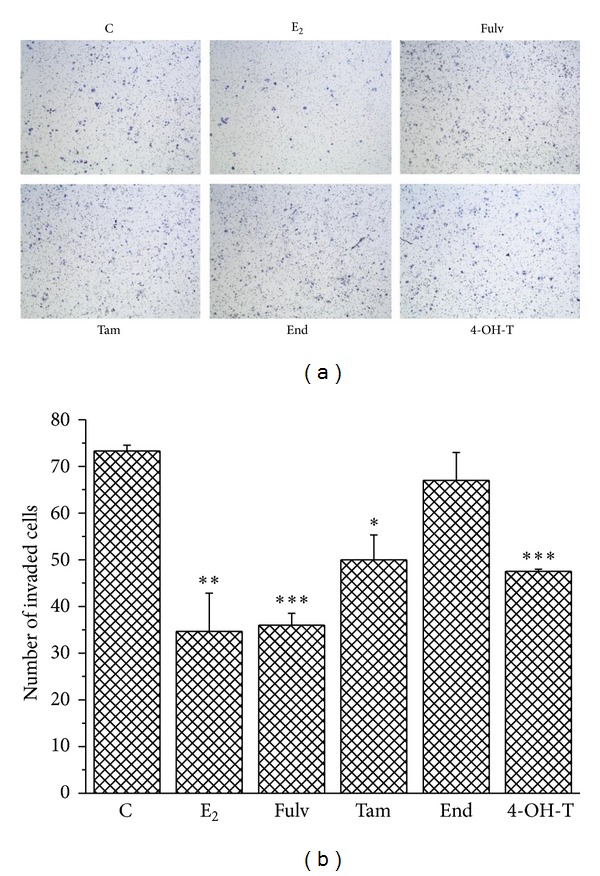
The effect of E_2_ and the tested agents on MCF-7 cell invasion. C: control (untreated cells); E_2_: cells treated with 17*β*-estradiol; Fulv: cells treated with E_2_ + 100 nM Fulv; Tam: cells treated with E_2_ + 100 nM Tam; End: cells treated with E_2_ + 100 nM End; and 4-OH-T: cells treated with E_2_ + 100 nM 4-OH-T. The image is representative of three independent experiments using a magnification of 10X (a). Quantification of images from boyden chamber assay in MCF-7 cells (b). Results are expressed as mean ± SEM of the % change compared to the untreated cells. Asterisks denote a statistically significant difference compared to untreated cells. *P < 0.05, **P < 0.01, and ***P < 0.001.

Because migration plays a crucial role during tumor invasion, we evaluated the influence of Fulv, Tam, and its active metabolites on the invasive capacity of breast cancer cells lines. Cell invasion was studied using a modified boyden chamber assay with a membrane coated with matrigel. Cells were treated with E_2_ and tested agents, and the invasion was observed 72 h later. In MCF-7 cells, we found that E_2_ alone reduced cell ability to invade and this effect was partially reversed by the combination of E_2_ and the tested agents (Figure 4). Fulv and 4-OH-T exerted a better inhibitory effect than Tam and End (Figure 4). T47D cells were not used in this set of experiments, because of the low capacity to migrate the membrane in typical boyden chamber assay. Although, MCF-7 cells are also characterized by low invasive capacity compared to other breast cancer lines, we showed that the treatment with E_2_ and the tested agents altered their motility and this prompted us to investigate it further.

### 3.5. Fulv and Tam Facilitate Invasion through MMPs' Modulation

**Figure 5:**
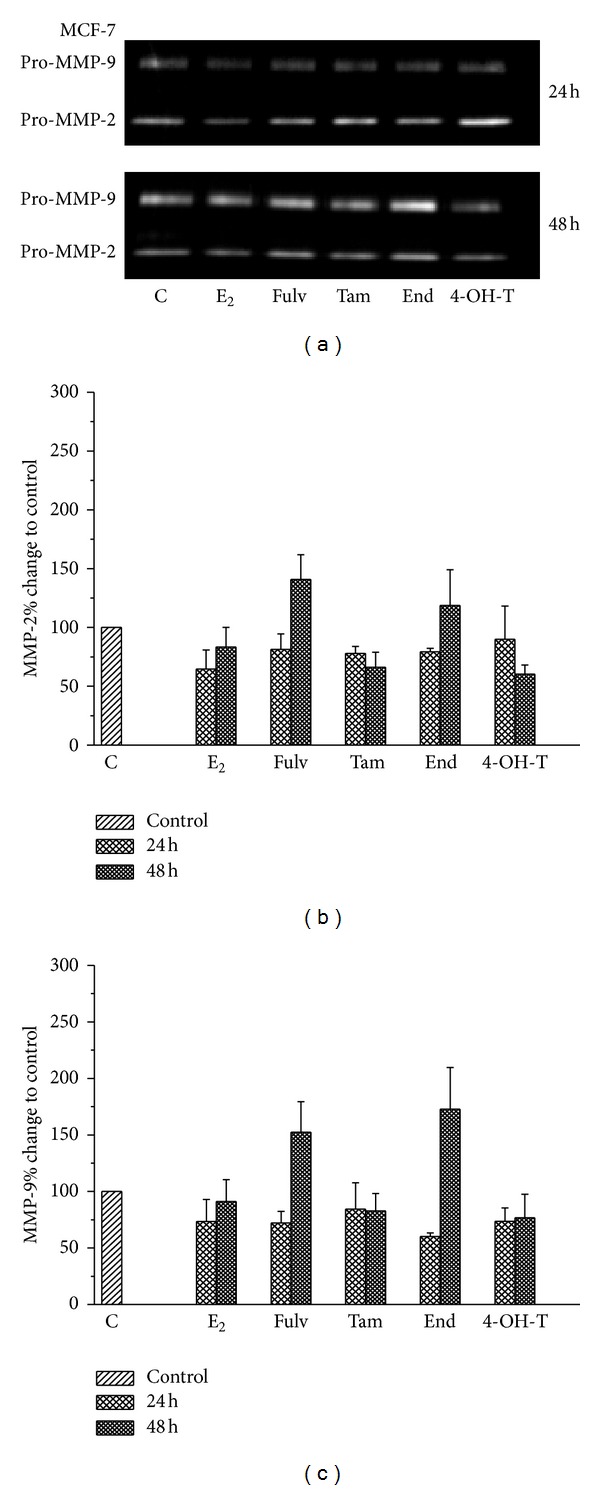
MMP-9 and MMP-2 enzyme expression after treatment of MCF-7 cells with E_2_ and the tested agents. (a) A representative image of three independent experiments. A quantitative analysis of images for (b) MMP-2 and (c) MMP-9 expression using appropriate software. Results are expressed as mean ± SEM of the % change compared to the untreated cells.

MMPs are key players in invasion and metastasis since they promote the invasive potential through digestion of the ECM components [5, 31, 32]. In ER^+^ breast tumors E_2_ exerts a protective role since it regulates the expression both of MMP-2 and MMP-9 as well as syndecan-4 [29] and, therefore, limits the ability of cells to invade the adjacent tissues. By contrast, antiestrogens seem to reverse this effect increasing the level of MMPs [33]. We evaluated the influence of E_2_ alone and/or in combination with the tested agents on MMP-2 and MMP-9 levels 24 and 48 h after treatment of cells. Zymography analysis in MCF-7 cells demonstrated a slight decrease on the expression of both MMP-2 and MMP-9 followed the treatment with E_2_ up to 48 h. In addition, the combination of cells with E_2_ and tested agents reversed the effect of E_2_ inducing MMPs levels 24 h after treatment of cells (Figure 5). This phenomenon was preserved for Fulv and End up to 48 h after cells treatment. At the same time point, when E_2_ combined with Tam, MMPs levels were not changed compared to E_2_ alone while the combination of E_2_ with 4-OH-T reduced the levels of MMPs and particularly MMP-9 (Figure 5). In T47D cells any change in MMPs levels was not found after cells treatment with E_2_ and the tested agents at any time point tested (data not shown).

### 3.6. Tam and End Stimulate EMT-A Different Role for Snail

**Figure 6:**
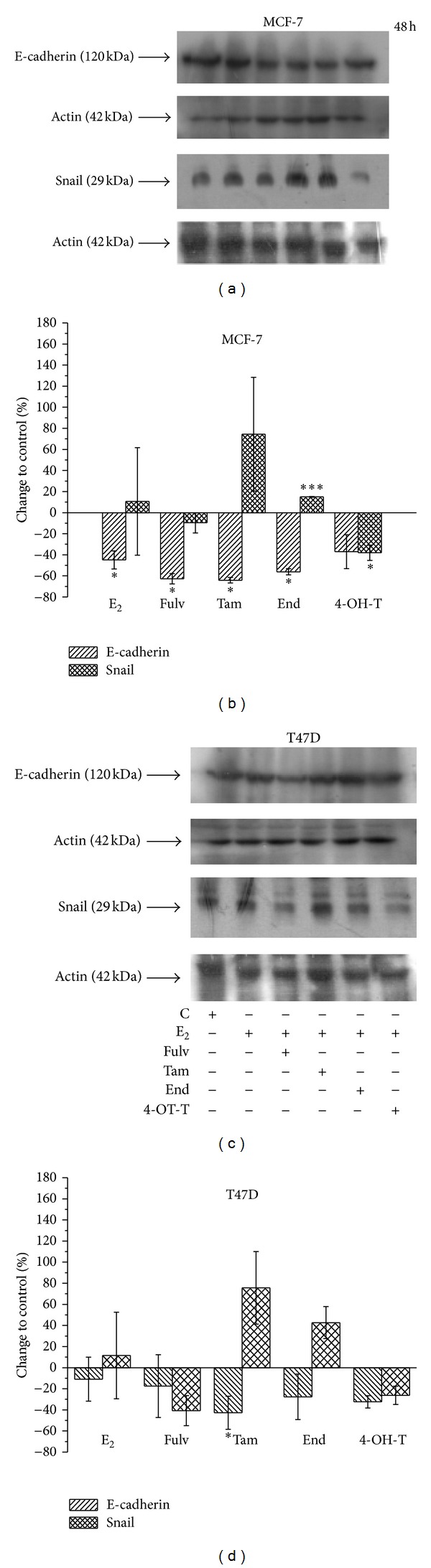
E-cadherin and Snail protein expression in MCF-7 and T47D cells 48 h after treatment of cells with E_2_ and the tested agents. A representative image of three independent experiments for both cell lines using western blot analysis, (a) and (c). Quantification of images from western blot analysis in both cell lines, (b) and (d). Results are expressed as % change compared to the untreated cells ± SEM. Asterisks denote a statistically significant difference compared to untreated cells. *P < 0.05 and ***P < 0.001.

At the leading edge of invasiveness and metastasis, epithelial cells undergo EMT. Two major partners of EMT are E-cadherin and Snail. E-cadherin is reversibly downregulated in EMT, and this reduction is associated with increased levels of Snail, a repressor of E-cadherin [28, 34, 35]. Regarding E-cadherin protein levels, we found that E_2_ alone and/or in combination with 4-OH-T did not alter protein status 48 h after their addition to MCF-7 cells. The combinations of E_2_ with Fulv, Tam, and End caused a decrease in E-cadherin protein levels (Figure 6(a)). Further, regarding Snail protein levels, we found that E_2_ alone increased Snail protein status at the same time point. The combination of E_2_ with Fulv and/or with 4-OH-T decreased Snail protein. This phenomenon was more potent in the case of E_2_ with 4-OH-T. The combination of E_2_ with Tam and End increased Snail levels (Figure 6(a)). In T47D cells, E_2_ alone as well as its combinations with Tam, End and 4-OH-T did not alter E-cadherin protein levels. The treatment of cells with E_2_ and Fulv caused a slight decrease in protein levels (Figure 6(b)). Furthermore Snail protein was decreased only when E_2_ combined with Fulv and 4-OH-T (Figure 6(b)).

The complicated results from western blot analysis revealed that, in both cell lines, the protein changes of E-cadherin did not follow the changes of Snail protein levels in order for an EMT phenomenon to be observed. Only in the case that E_2_ combined with Tam or End, a decrease of E-cadherin levels followed an increase of Snail levels. In addition, the most important changes were observed at Snail protein after cell treatment with the combinations of E_2_ with Fulv and 4-OH-T where Snail levels were decreased (Figure 6). Besides in EMT, the role of Snail is also very important for cell survival. Previous studies have shown that a decrease in Snail protein sensitizes cell to death [34, 36].

### 3.7. The Antiestrogens on Localization of E-Cadherin and Snail

**Figure 7:**
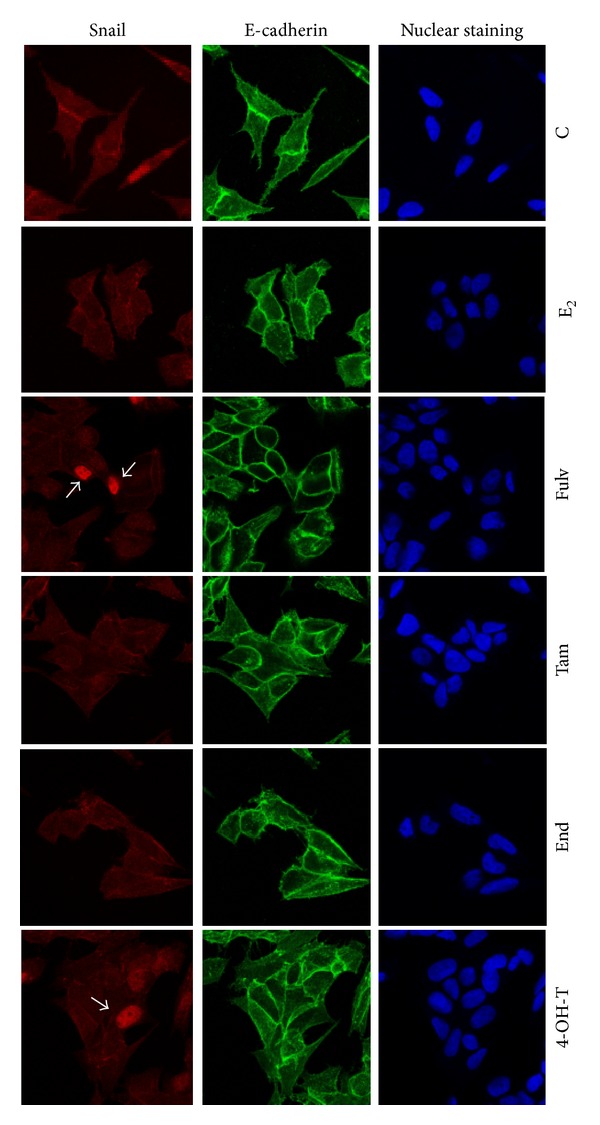
E-cadherin and Snail protein localization in MCF-7 cells 48 h after treatment of cells with E_2_ and the tested agents. C: control (untreated cells); E_2_: cells treated with 17*β*-estradiol; Fulv: cells treated with E_2_ + 100 nM Fulv; Tam: cells treated with E_2_ + 100 nM Tam; End: cells treated with E_2_ + 100 nM End; and 4-OH-T: cells treated with E_2_ + 100 nM 4-OH-T. The image is representative of three independent experiments using a magnification of 63X.

In order for the transcription factor Snail to act as repressor of E-cadherin, its nuclear translocation is required. Since western blot analysis did not reveal any significant connection between EMT proteins' expression and treatment of cells with the E_2_ and the tested agents, we studied the effect of antiestrogens on these proteins' localization, 48 h after cell treatment. We found that E-cadherin is located in cell membrane and cell-cell junctions in untreated MCF-7 cells as well as in cells treated with E_2_ and the tested agents (Figure 7). Snail was localized at both nucleus and cytoplasm in untreated cells or cells treated with E_2_ (Figure 7). The combinations of E_2_ with Fulv and 4-OH-T retained the cytoplasmic localization and enhanced the nuclear localization. The combinations of E_2_ with Tam and End retained the cytoplasmic localization of Snail. Similar effects of E_2_ and the tested agents were observed at T47D cells (data not shown).

### 3.8. Fulv and Tam Affect Migration through FAK Phosphorylation and F-Actin Rearrangement

**Figure 8:**
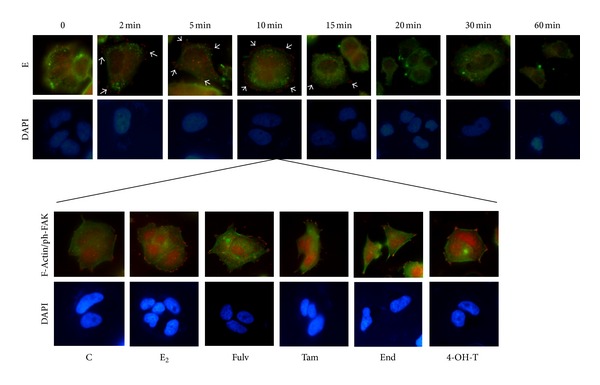
The impact of E_2_ and the tested agents on Tyr^397^ FAK phosphorylation and F-actin rearrangement. MCF-7 cells exposed to E_2_ in a time course manner up to 60 min for detection of the maximum FAK phosphorylation. ER*α* and Tyr^397^ FAK localisation is indicated with green and red fluorescence, respectively. At the time point of 10 min, F-actin and Tyr^397^ FAK colocalisation (green and red fluorescence, resp.) was observed after the exposure of MCF-7 cells to the tested agents. C: control (untreated cells); E_2_: cells treated with 17*β*-estradiol; Fulv: cells treated with E_2_ + 100 nM Fulv; Tam: cells treated with E_2_ + 100 nM Tam; End: cells treated with E_2_ + 100 nM End; and 4-OH-T: cells treated with E_2_ + 100 nM 4-OH-T. The image is representative of three independent experiments using a magnification of 60X.

FAK exerts a central role on cell migration and invasion, and its activation is correlated with malignant transformation [37, 38]. In addition, a specific phosphorylation at Tyr^397^ residue is correlated with Tam-resistance [22]. In MCF-7 cells, E_2_ exposure resulted in autophosphorylation of FAK in Tyr^397^ residue, which entails activation of FAK. This phenomenon was time dependent, and the highest phosphorylation was observed in 10 min (Figure 8). Thereafter, the phosphorylated signal was downregulated.

At the time point of 10 min, when the maximum FAK phosphorylation was found, we investigated the impact of Fulv, Tam, and its metabolites in spatial organization of actin fibers. The main finding to emerge was that the treatment of cells with E_2_ combined with Fulv either Tam or End resulted in a less round-like morphology with more leading edges than the other groups (Figure 8). The colocalisation of F-actin with Tyr^397^ FAK appeared mainly at the leading edges. In untreated cells as well as in cells treated with E_2_ alone or in combination with 4-OH-T, the spots of Tyr^397^ FAK are scattered all around the cell membrane which is attributed to increased stability (Figure 8). Similar effects of E_2_ and the tested agents were observed at T47D cells (data not shown).

## 4. Discussion

Hormonal therapy has been established for the treatment of ER^+^ breast cancer patients. Several clinical trials [39–41] have demonstrated the benefits of this type of treatment, and it is generally acceptable that it has contributed to the decrease in breast cancer mortality. Despite the benefits of hormonal therapy, the disease often relapses and secondary tumors develop due to their metastatic potential [42, 43]. *In vitro* studies have assessed the impact of antiestrogens on breast cancer cell invasiveness and MMPs expression [16, 33, 44, 45]. In the present study we evaluated the effect of the antiestrogens Fulv and Tam from a different standpoint, namely, migration that leads to tumor growth, invasion, and metastasis.

There are many types of cell movement that lead to cell migration and invasion according to cell type and microenvironment [4]. Epithelial cells undergoing EMT can migrate individually. On the other hand, basal- and squamous-originated epithelial cells following EMT or moderately differentiated epithelial cells lacking EMT can migrate collectively [4]. In order to evaluate the effect of E_2_ on single and collective cell migration, we applied 2 typical assays: boyden chamber and wound healing, respectively. We found that in MCF-7 cells, E_2_ alone failed to stimulate single cell migration while promoting collective cell migration in both cell lines. The failure of E_2_ to stimulate single cell migration is in line with the unclear results of western blot analysis for the interaction of EMT proteins, E-cadherin, and Snail as well as with the absence of Snail import to the nucleus. Snail is a highly unstable protein and is dually regulated by protein stability and cellular localization. In order for Snail to exert its effect, a nuclear translocation is required [34]. The increase in collective cell migration after treatment of cells with E_2_ is in line with the increase in cell proliferation of both cell lines since these are indications of expansive growth with the absence of active migration [46]. In contrast to the increase in cell proliferation and collective cell migration, we found that E_2_ decreased the capacity of cells to invade. The decrease in invasiveness was associated with decrease in MMPs. This is not the first time that a protective role of E_2_ is described. Previous studies have shown that E_2_ may inhibit breast cancer cell invasion by affecting proteins that modulate cell-cell interactions or increasing the number of desmosomes [47]. The reduced invasiveness of E_2_-stimulated cells is also supported by the findings from immunofluorescence assay, where cells demonstrated a more spherical morphology with focal adhesions all a round the cell membrane, which is associated with increased stability.

Using Fulv, the mitogenic effect of E_2_ was partially reversed with a decrease in Snail protein levels associated with its import to nucleus. However, the effect of E_2_ either on single or collective cell migration was not altered. Fulv is a selective estrogen downregulator that binds to ER forming an unstable ER-Fulv complex, which is rapidly degraded resulting in ER reduction. Fulv may exert genomic as well as non genomic effects on target cells [16, 48]. A recent publication by Song et al. [48] shows that Fulv at the concentration of 0, 1 *μ*M shuttles ER*α* from the nucleus to the cytosol and plasma membrane. When Fulv is extra-nuclear acts as an estrogen agonist but after its entrance to the nucleus blocks the genomic effects of estrogens in transcription and cell proliferation. This might explain the effect on cell proliferation but not on cell migration. Previous data have shown that functional ER*α* is associated with E-cadherin expression, and this expression as well as cell-cell adhesion may be modulated by antiestrogens resulting in an invasive phenotype [16]. Indeed, we found that Fulv decreased E-cadherin protein expression and increased cell invasion and MMPs expression versus E_2_. These data were confirmed by immunofluorescence assay where cells exhibited a less round-like morphology, indication of increased invasiveness.

Tam is a prodrug that is metabolized to End and 4-OH-T so as to exert its therapeutic effect. Although both metabolites are equivalent regarding ER*α* binding and inhibition of E_2_-induced cell proliferation, it is proposed that End is the principal antiestrogenic metabolite for the antitumour activity observed in breast cancer patients [49]. In the current study we used both metabolites to verify that they act in the same way. Tam and its metabolites stimulated single cell migration and reduced collective cell migration. Regarding Tam and End, the stimulation of single cell migration is in concordance with the E-cadherin protein decrease and Snail protein increase. This might be an indication that an active migration through EMT induction occurs after Tam and End treatment. Although Snail was not detected to the nucleus at the same time point we cannot exclude a positive role of cytosolic Snail in cell migration [50]. These data are also in agreement with the less round-like shape of cells as well as with the scattering of focal adhesions at the leading edges of F-actin revealing a more invasive and potent phenotype. An increase in both MMPs expression and cell invasiveness might facilitate EMT induction. In the case of 4-OH-T, it seems that an EMT phenomenon did not occur because no decrease in E-cadherin or increase in Snail protein levels was detected. In contrast, a reduction in Snail protein in association with a nuclear localization was detected. So far, our data indicate that 4-OH-T promoted single cell migration without EMT. A detailed review of Friedl and Alexander [4], related to the types of cancer cell movement, referred to a single cell movement currently known as amoeboid migration. In this type of migration, cells adopt a more spherical shape and migrate without ECM proteolysis. The decrease in MMPs levels and the spherical shape of cells found in our study after treatment with 4-OH-T using zymography and immunofluorescence, respectively, support this type of migration. The decrease of Snail protein and its nuclear location after 4-OH-T treatment seem to correlate with the inhibition of cell proliferation rather than migration. This is compatible with the decrease of cell proliferation that we found after 4-OH-T treatment. This decrease was more potent for 4-OH-T compared to the other agents which did not reduce Snail protein. Regarding invasion, it seems that the active single cell migration with or without EMT was associated with increased invasiveness.

## 5. Conclusions

**Figure 9:**
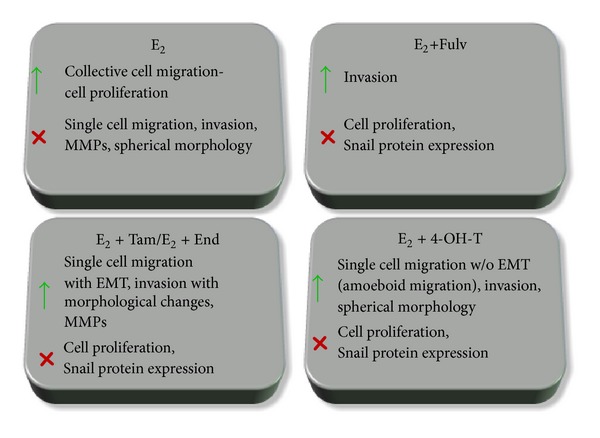
An overview of the effect of Fulv, Tam, and metabolites of Tam in migration and invasion of MCF-7 and T47D cells.

Our working hypothesis was that different approaches of estrogen inhibition affected differently breast cancer cell migration and invasion. Summarizing our data, we may conclude that in breast cancer cells after serum E_2_ withdrawal (i) E_2_ stimulated expansive growth of cells with the absence of EMT but exerted a protective effect by reducing invasiveness, MMPs expression and preserving a more stable phenotype with focal adhesions all around the cell membrane; (ii) the antiestrogens partially counteracted the E_2_-induced effect; (iii) Fulv did not affect the expansive growth stimulated by E_2_ and promoted cell invasion; (iv) Tam and its metabolites stimulated active single cell migration and increased cell invasiveness. An overview of Fulv and Tam effect is observed in Figure 9.

Although Fulv might result in a less aggressive behaviour of cells compared to Tam, the benefits of hormonal therapy concerning invasion and metastasis yet remain under question.

## Abbreviations

ER: Estrogen receptor
Fulv: Fulvestrant
Tam: Tamoxifen
E2: 17*β*-Estradiol
End: Endoxifen
4OHT: 4-OH-Tamoxifen
MMPs: Matrix metalloproteinases
SERDs: Selective estrogen receptor downregulators
ECM: Extracellular matrix
EMT: Epithelial mesenchymal transition
FAK: Focal adhesion kinase
FBS: Fetal bovine serum
rf-RPMI: Phenol red-free RPMI
CSS: Charcoal-stripped serum
MTT: 3-(4,5-Dimethylthiazol-2-yl)-2, 5-dephenyltetrazolium-bromide.
